# Spinal Cord Organoids to Study Motor Neuron Development and Disease

**DOI:** 10.3390/life13061254

**Published:** 2023-05-25

**Authors:** Felix Buchner, Zeynep Dokuzluoglu, Tobias Grass, Natalia Rodriguez-Muela

**Affiliations:** 1 German Center for Neurodegenerative Diseases, 01307 Dresden, Germany; felix.buchner@dzne.de (F.B.); zeynep.dokuzluoglu@dzne.de (Z.D.); tobias.grass@dzne.de (T.G.); 2 Center for Regenerative Therapies Dresden, Technische Universität Dresden, 01307 Dresden, Germany; 3 Max Planck Institute for Molecular Cell Biology and Genetics, 01307 Dresden, Germany

**Keywords:** spinal cord (SpC), motor neuron (MN), development, in vitro disease modeling, induced pluripotent stem cells (iPSCs), motor neuron diseases (MNDs), organoids, spinal cord organoids (SCOs)

## Abstract

Motor neuron diseases (MNDs) are a heterogeneous group of disorders that affect the cranial and/or spinal motor neurons (spMNs), spinal sensory neurons and the muscular system. Although they have been investigated for decades, we still lack a comprehensive understanding of the underlying molecular mechanisms; and therefore, efficacious therapies are scarce. Model organisms and relatively simple two-dimensional cell culture systems have been instrumental in our current knowledge of neuromuscular disease pathology; however, in the recent years, human 3D in vitro models have transformed the disease-modeling landscape. While cerebral organoids have been pursued the most, interest in spinal cord organoids (SCOs) is now also increasing. Pluripotent stem cell (PSC)-based protocols to generate SpC-like structures, sometimes including the adjacent mesoderm and derived skeletal muscle, are constantly being refined and applied to study early human neuromuscular development and disease. In this review, we outline the evolution of human PSC-derived models for generating spMN and recapitulating SpC development. We also discuss how these models have been applied to exploring the basis of human neurodevelopmental and neurodegenerative diseases. Finally, we provide an overview of the main challenges to overcome in order to generate more physiologically relevant human SpC models and propose some exciting new perspectives.

## 1. Introduction

The spinal cord (SpC) plays the crucial role of transmitting signals between the brain and the peripheral body, including the relay of sensory information and motor instructions, reflex actions and the generation of motor activity [1]. A variety of cell types, interneurons and motor neurons (MNs)—cholinergic neurons found in the ventral horns of the SpC and the brainstem—are involved in these motor functions. The anatomical arrangement of spinal MNs (spMN) within the SpC correlates with the muscles they innervate. According to this, spMNs are organized in spMN pools, a cohesive group of spMNs with similar intrinsic properties that are anatomically arranged to connect with one singular target muscle in the periphery [2]. spMNs receive innervation from upper MNs situated in the motor cortex of the brain, directly or after one or multiple relays in spinal interneurons. spMNs can be also classified according to different features, such as their morphology and size of axonal projections, firing rate or based on the rostro–caudal segment of the SpC where they are located [3]. Based on this, spMNs are divided as: branchial, found in the brainstem and responsible for controlling facial and neck muscles; visceral, innervating a part of the autonomic nervous system and regulating smooth muscle; and somatic, which innervate skeletal muscles [3]. 

Most of our knowledge on spMN diversity and biology as well the SpC development was generated from in vivo studies in model organisms. However, the advancement in the last decade of in vitro models to study human development and disease is enabling scientists to tackle questions that were difficult to address in vivo. In vitro-generated three-dimensional tissue-like models are pluripotent stem cell (PSC)-derived complex systems arising from the self-organization of multiple cell types, with distinct cytoarchitecture and that partially recapitulate the functional features of an organ. These tissue-like structures are classified under the umbrella term organoids (ORG) [4,5]. Spheroids constitute a more simplistic tissue-like model that is typically composed of homogenous cellular types and obtained by combining one or more separately patterned cell types that have limited self-organization properties in a 3D culture [6]. Nervous system ORGs are named after the specific anatomical region they mimic, such as cortical, retinal, hypothalamic or SpC organoids (SCOs). In 2013, Sasai’s group reported pioneering findings on the self-organizing capabilities of human embryonic stem cells (hESCs) when cultured in specific floating conditions, which included axial polarity, inside–out layering, patterning and curving morphology, driven by internal programs characteristic of the human neocortex [4]. Around the same time, Knoblich’s team developed the first 3D culture model mimicking human brain development and coined the term “cerebral organoid” [5]. By applying scRNAseq technology, Camp and colleagues demonstrated shortly afterward that such human-induced pluripotent stem cell (hiPSC)-derived cerebral organoids closely recapitulated the gene expression programs driving neocortex development in the human fetus, which ultimately led to the generation of a first cell atlas of human corticogenesis [7,8]. 

Despite our extensive understanding of the anatomy of the SpC, its cellular composition, neuronal circuitry and embryological development, many fundamental questions remain to be resolved. How do extracellular signals regulate the expression of lineage-allocating transcription factors (TFs) during spMN development and instruct the signaling state of regulatory mechanisms that maintain this developmental fate? How can we manipulate these signals to direct the differentiation of specific MN subtypes? How is the layered organization of the SpC achieved on a molecular level? Can abnormalities in human SpC development underlie or contribute to the emergence of neurodegenerative diseases (NDs)? To address these and other questions, in the last decade, the scientific community has made use of stem cell (SC)-derived in vitro approaches as a valuable complementary asset to in vivo studies. While an extensive number of reports have kept optimizing protocols for the generation and study of cortical organoids, SCOs have been significantly less pursued. However, SCOs have emerged as a useful resource for studying human SpC development in recent years. In this review, we will outline the SpC and spMN development and summarize the methods used to produce such cultures, their evolution and the current state-of-the-art approaches that have significantly contributed to the study of SpC development and the modeling of the most common neuromuscular disorders such as spinal muscular atrophy (SMA) and amyotrophic lateral sclerosis (ALS). Furthermore, we will discuss how closely these approaches recapitulate human physiology and pathology and what recent advances and challenges exist in the field to improve the formation of human SpC-like structures in vitro.

## 2. Spinal Cord Organoids (SCOs): Evolution of In Vitro Protocols for Recapitulating Spinal Cord Development

### 2.1. Morphogen Gradients Direct SpC Development and Emergence of spMNs

**Figure 1:**
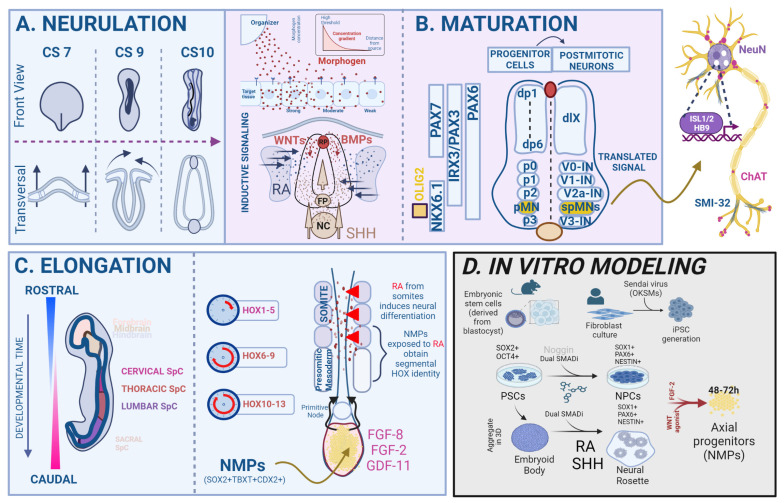
Spinal cord (SpC) organogenesis and recapitulation in vitro. (**A**) During neurulation—depicted from several Carnegie stages, CS—the neuroectoderm folds and builds a tubular structure that will later develop into the SpC. (**B**) Inductive signaling from molecular organizer regions, which secrete morphogens, causes the primitive neural tube to be patterned across the DV axis. This patterning is translated into domain-specific expression of transcription factors creating neuronal diversity and heterogeneity. (**C**) At the same time, the embryo undergoes body column elongation from rostral to caudal, causing the emergence of SpC axial segments. When the neuromesodermal progenitors (NMPs) located in the tail bud are exposed to retinoic acid (RA), released from the adjacent somites, they start to differentiate and adopt colinear HOX expression. (**D**) In vitro, hiPSCs can be patterned into neural progenitor cells (NPCs)—either in 2D or aggregated in an embryoid body (EB)—through dual SMAD inhibition and may alternatively be induced to differentiate into axial progenitors through WNT-FGF signaling. Created with BioRender.com (accessed on 2 May 2023).

By the time of a late gastrulation, germ layer separation in the vertebrate embryo is nearly complete, and multipotent cells in the epiblast have started to differentiate into lineage-restricted progenitor cells. Concomitantly, neural induction occurs and neuroepithelial cells form a sheet-like neural plate, which undergoes a series of rapid morphological changes (convergence–extension–elevation–fusion) to become the primitive neural tube. This process occurs in two modules, whereby primary neurulation forms the brain and rostral SpC, while secondary neurulation contributes to the caudal SpC (Figure 1A). Moreover, the SpC undergoes continuous growth during embryogenesis, termed axial elongation, which results in the formation of four distinct anatomical regions along the rostro–caudal (RC) axis, namely the cervical, thoracic, lumbar and sacral SpC segments [9]. As the SpC develops, new cells are continuously generated from a proliferating posterior growth zone called the tail bud, a transient structure located at the caudal end of the embryo composed of undifferentiated cells with SC-like properties. These cells, called neuromesodermal progenitor (NMPs), are a bipotent cell population capable of self-renewal that only exists during the embryonic phase and is characterized by a co-expression of the TFs *TBXT* and *SOX2* [10,11]. A tightly regulated cascade of wingless and integrated (WNT) and fibroblast growth factor (FGF) signaling maintains the NMPs in an undifferentiated progenitor state [12,13]. The capacity of NMPs to contribute to multiple germ layers was proven by performing engraftment experiments in chicken and GFP mouse embryos. The resulting chimeric organism showed that cells in the transplanted NMPs contributed to both neural and mesodermal sub-lineages in the host embryo, especially in the lumbar region of the SpC and its surrounding paraxial mesoderm [14,15,16]. Lineage-tracing experiments by Gouti and colleagues helped to characterize the NMP transcriptome, providing the final proof that these cells indeed gave rise to both the SpC and caudal embryonic muscles [17,18]. It remains a subject of discussion, however, whether all NMPs first acquire a specific axial identity and afterward commit to a neural identity or vice versa [19]. 

One of the most intriguing questions about the development of the vertebrate SpC is by which mechanisms the correct size, proportion and anatomical architecture are established from a primitive tissue layer, the neural tube. Over the past thirty years, studies have shown that several morphogens diffuse from the dorsal and ventral poles of the developing SpC and seemingly instruct this process. Per definition, such morphogens are diffusible molecules that can form concentration gradients within a tissue. Cells can sense their position within these gradients to determine their developmental fate by precisely regulating their gene expression in a spatially delimited area [20]. The translation of the morphogen signals into gene regulatory networks (GRNs) relies on the cross-regulation of TF activity, allowing for the integration of incoming morphogen signaling dynamics [20]. During the development of the SpC, morphogens that belong to the bone morphogenetic protein (BMP) and WNT families are secreted by the embryonic roof plate (RP) and instruct dorsal progenitor identity, whereas Sonic hedgehog protein (Shh), dissipated from the notochord and floor plate (FP), specifies a ventral identity [21,22,23,24]. The long-range diffusion of Shh induces the differentiation of ventral interneurons and spMNs at specific positions in the ventral SpC according to a specific dorsoventral (DV) pattern [23], and accordingly, its loss causes the absence of ventral cell types and the expansion of the dorsal progenitor domain [25,26]. The embryonic SpC is divided into discrete, dorsoventrally assembled progenitor domains, which are characterized by a unique profile of TF expression. There are five SpC progenitor domains (p0–p3 and pMN), which develop into either interneurons (V0–V3) or spMNs (pMN) [9] (Figure 1B).

Downstream of Shh signaling, the progenitor MN (pMN) domain is established and initially defined by the co-expression of the neuroectodermal TFs *PAX6* and *NKX6.1*. Within this domain, the pMN marker OLIG2 is induced by *NKX6.1*, and specifies a pan-spMN fate [27,28]. Recent single-cell transcriptomic studies have shed light on the transcriptional changes that occur during the transition from pMNs to mature spMNs, whereby *OLIG2* represses *HES* TFs (such as *HES1* and *HES5*), which are NOTCH pathway effectors and act as neurogenesis antagonists. Ultimately, high *OLIG2* and *PAX6* levels induce the expression of the proneural TF *NEUROG2*, which dictates the timing of differentiation by marking progenitor cells for cell cycle exit and further development into immature, postmitotic spMNs [29]. Newly differentiated spMNs express the TF *HB9* shortly after exiting the cell cycle [30]. As differentiation progresses, spMNs acquire the expression of *ISL1* and specify into different subtypes along the RC axis [31]. Other TFs belonging to the LIM homeodomain family are also expressed in postmitotic spMNs, such as *LHX3* and *LIM1*, contributing to their molecular identity [32,33]. The expression of spMN-associated TFs, such as *ISL1/2* and *HB9* and appearance of filamentous, cytoplasmic maturity markers (SMI32, vChAT) or nuclear antigens such as NeuN, allows for the identification of spMNs. Recent studies have investigated the spatial and temporal TFs expression dynamics during mouse SpC embryogenesis at single-cell resolution [34]. Additionally, several scRNAseq reports on fetal and adult human SpC have provided a comprehensive assessment of the TFs that encode spMN identity and generated SpC atlases [35,36,37].

At the molecular level, the axial identity of NPCs is heavily influenced by caudalizing morphogens that trigger the expression of a group of TFs called homeobox (*HOX*) genes [38,39]. These genes are highly evolutionarily conserved and play a critical role in consolidating the genetic hierarchy of developmental patterning modules across phylogenic species [40]. The main function of *HOX* genes, which are expressed in overlapping patterns along the RC axis of various developing tissues, is to sequentially provide an anterior–posterior identity within the developing body column, precisely encoded on a genetic level [9,41]. Often, this pattern is referred to as the “HOX code” of a body segment [42]. The importance of HOX patterning in the development of spMNs has been shown in vivo through controlled genetic changes in *HOX* gene expression boundaries and inactivation studies in mice. The former manipulation led to segmental identity alterations and homeotic transformations [43]. Taking the second approach, Lin et al. showed that the loss of lumbar *HOXA10* and *HOXD10* genes resulted in a significant reduction in the number of medial and lateral spMNs in the lumbar SpC [44]. Intense investigation is ongoing to understand the mechanisms underlying the establishment and maintenance of *HOX* gene expression. Mechanistically, the expression of *HOX* genes is regulated by gradients of WNT and FGF signaling during the anterior spreading of the primitive streak [45,46]. Specifically, *HOX* genes expressed in more rostral parts of the SpC are induced by lower amounts of FGF, whereas the *HOX* expressed in a more caudal part are induced by higher amounts of FGF [45]. Importantly, this code can be, at least partially, replicated in vitro [47,48,49]. Evidence from in vitro and in vivo experiments suggests that retinoic acid (RA) is another morphogen that plays important roles in organogenesis and the differentiation of the brain and SpC, for instance, by inducing expression of *HOX* genes [50,51,52] (Figure 1C). Once the SpC is specified, RA release from the adjacent somites refines the positional identity of neurons along the RC axis. RA interacts with RAR and RXR receptors, causing heterodimers that bind to RA response elements (RAREs) in promoter regions of *H*OX*ox* genes [53,54,55]. The connection of axial elongation with RA signaling is likely, as studies conducted in quail embryos unveiled that a lack of RA signaling leads to abnormalities in the RC organization of the embryonic SpC [56,57,58]. Additionally, studies on the mechanisms assessing hindbrain segmentation in mice revealed that *HOX* genes play a role in regulating components of RA signaling, which support feedback loops that strengthen the crosstalk between *HOX* gene expression and RA signaling [59]. 

In summary, the specificity in SpC circuitry formation is achieved through the spatial segregation of functionally distinct cell types at predetermined locations, with the position of a progenitor cell being a primary determinant of its developmental fate. Morphogens secreted from different cell sources are distributed in the developing embryo, with RA, secreted from the somites and FGFs from the tail bud region, playing a role in patterning the SpC along the RC axis, while BMP/WNT and SHH, secreted from the RP and FP, respectively, contribute to patterning along the DV axis. Only the perfectly tuned orchestration of these processes ensures the correct development of this highly complex nervous tissue. A comprehensive overview of the transcriptional mechanisms governing spMN specification is compiled in [60].

### 2.2. Two Roads to Generating spMNs In Vitro: Guided Differentiation and Transcriptional Programming by TF Overexpression

Both embryonic stem cells (ESCs) and human-induced pluripotent stem cells (hiPSCs)-derived MN cultures have been widely used in the last two decades for investigating spMN biology in health and disease. Whereas ESCs are derived from the inner cell mass of the mammalian blastocyst, iPSCs are created by genetically reprogramming somatic cells, such as fibroblasts. This is achieved by overexpressing the “Yamanaka TFs”, *OCT4*, *SOX2*, *KLF4* and *C-MYC* [61]. These PSCs can be differentiated into spMNs by using specific small molecules that mimic the signaling factors involved in SpC development in vivo or by directly overexpressing spMN lineage-allocating TFs [62] (Figure 1D). 

Most in vitro protocols for the guided differentiation of PSCs into neural cells follow the developmental rationale of the activation–transformation model of neural induction [63]. This model proposes an initial rapid PSC induction toward anterior neuroectoderm through inhibition of the TGFß/BMP signaling pathway, followed by the acquisition of more posterior identities through the exposure of neural stem cells (NSCs) to caudalization signals, such as RA or FGF8. To generate high quantities of NSCs/NPCs from PSCs, it is crucial to precisely understand how to “activate” neuroectoderm formation in order to gain control of the neural induction process. A plethora of protocols has been described to achieve this. Aggregation of PSCs in suspension cultures, resulting in 3D structures known as spheroids or embryoid bodies (EBs), triggers the spontaneous formation of various cell types, including neuronal cells [64]. However, unguided differentiation of PSCs into EBs was inefficient and resulted in only a few cells expressing NSC markers, such as SOX1, and a minimal expression of neuronal markers, such as NEUN [65]. Studies by Vallier and colleagues were pivotal in the improvement of neural differentiation protocols, as they demonstrated the importance of inhibiting the TGFβ-signaling effector Nodal in hESCs to activate neuronal specification and the expression of the proneural gene *NEUROD1* [66]. Shortly afterward, it was shown that culturing hESCs in N2/B27 medium supplemented with the BMP antagonist Noggin led to the generation of NESTIN + NPCs contained within neural rosettes [67]. Another study discovered that treatment of hESCs with the small molecule SB431542 (SB), the inhibitor of the TGF-β/Activin/NODAL pathway, enhanced the neural induction processes [68]. Although studies up to that point emphasized that treatment with Noggin or SB alone induced neuroectoderm formation from hESCs in both 2D and EB cultures, the efficiency of such induction was limited. Later reports demonstrated that using SB and Noggin in combination to achieve a simultaneous inhibition of the TGF-β and BMP signaling pathways (called dual SMAD inhibition) on hESC monolayer cultures, resulted in a more efficient and rapid generation of stable neuroectoderm, with over 80% PAX6+; SOX1 + NPCs after 4–8 days, a swift disappearance of the PSC markers OCT4 and NANOG and increased expression of the proneural marker genes [69] (Figure 1D). Later, Surmacz and colleagues sought to replace the commonly used recombinant protein Noggin by the small molecule BMP inhibitor LDN193189 (LDN) and reported that small molecule-based dual SMAD inhibition with LDN/SB for only 1 day was sufficient to efficiently generate PAX6 + NSCs from PSCs [70]. Together, these findings demonstrated the importance of investigating the mechanisms of neural induction, as they empowered the development of more efficient and controlled protocols for guiding PSC differentiation toward neural lineages. 

After neural induction from PSCs was achieved, most protocols aimed at generating spMNs or SpC-like tissue exploring different routes to further guide cell specification toward the acquisition of a specific RC fate. This was possible by exposing NSCs to RA and SHH to replicate the developmental signals emerging from the somites (RA) and FP/notochord (SHH) during neural tube development in vivo [9]. In the first study that reported the production of ISL1+ spMNs from mouse ESCs, Wichterle and colleagues demonstrated that RA for 2–3 days together with SHH agonist promoted hindbrain/cervical SpC fate. After 5 days in vitro, a substantial number of cervical, *HOXC5*-expressing ISL1+ neurons were detected, evidencing a successful acquisition of rostral spinal positional fate [65]. While earlier studies had already demonstrated that RA treatment of mESCs-derived EBs induced expression of the spMN marker ISL1 at very low yields (2–3% of all cells) [71], Wichterle and colleagues pioneered the refinement of protocols that yielded 20–30% of HB9: eGFP + spMNs [65,72]. Importantly, the spMN progenitors generated through this protocol showed efficient spMN differentiation and engraftment when transplanted into chick embryos. Subsequent studies were conducted to transfer this knowledge to a human context. Li and colleagues were the first to adapt the protocol previously established by Wichterle on mouse ESCs to hESCs, achieving a similar yield of ~20% spMNs [73]. Later studies conducted a functional characterization of hiPSC-derived spMNs using patch clamp recordings, which revealed their repetitive firing in response to stimulation [74]. Extensive research since those early days was devoted to improve spMN differentiation from hiPSC/hESCs by adjusting the timing, length and concentrations of these SpC morphogens, their combination with additional patterning molecules and the culturing media [75,76,77]. 

Notably, there is some controversy regarding the importance of RA-mediated signaling to induce spMN differentiation from PSCs. Patani and colleagues showed that inhibiting TGFβ/Activin/Nodal signaling with SB and later adding a SHH agonist (SAG) was able to specify PSCs into spMN precursors even in the presence of RA pathway antagonists [78]. This method yielded a reduced but consistent percentage of OLIG2 + pMNs and HB9 + spMNs, demonstrating that the development of spMNs in vitro was at least partially independent of RA pathway activation. 

In addition to TGFß/BMP/RA and SHH signaling pathways, the activity of NOTCH is often modulated to accelerate the differentiation of NPCs into specific neural sub-lineages, such as spMNs. γ-secretase-mediated inhibition of the NOTCH signaling pathway through DAPT during early NPC emergence from msESCs led to the increased formation of OLIG2 + pMNs and 20% more HB9+; SMI32 + spMNs [77]. Importantly, the acceleration of spMN development through DAPT in vitro did not seem to affect axial identity or induce aberrant lineage allocation [48]. Similarly, the effect of pulse-wise NOTCH inhibition at late stages of spMN in vitro differentiation proved effective at increasing the amount of *ISL1* expression by more than 5-fold [79]. Based on the existing literature, it is evident that two distinct trends have emerged in the field of in vitro spMN generation. The first trend focuses on optimizing the conditions for efficient neuroectoderm formation, while the second trend aims to direct the neurogenic fate toward ventral SpC cell lineages, utilizing RA to induce caudalization. Finally, it is worth mentioning that 3D-based methods for guiding the differentiation of hiPSC into neuronal lineages have proven a higher efficacy when compared to analogous 2D-based approaches. Comparative studies have revealed that 3D neural induction resulted in a higher number of PAX6 and NESTIN expressing cells, along with more complex neurite arborization in the derived neurons and increased astrocyte formation [80,81]. 

An alternative approach to these developmentally inspired guided differentiation protocols relies on the induction of specific TFs to directly convert somatic cells into MNs or to transcriptionally program PSCs into MNs or bypassing developmental stages. Although this approach was intensively explored in the past, and proved advantageous especially to model late-onset diseases, given that the epigenetic clock of the donor cells does not reset during cell reprograming, its limited efficiency and reduced scalability turned many researchers to the second alternative for directed differentiation (reviewed in [62]). It is worth noting that spMNs formed by the transcriptional programming of PSCs or lineage conversion do not express NPC *SOX1* and *OLIG2* TFs, which raises concerns with regard to the fidelity of modeling embryological development in vitro when utilizing these approaches [82]. Additionally, these methods may produce cell types with subtle defects resulting from their atypical developmental histories [83]. However, a report showed that using the same mouse ESCs as a starting cell source, spMNs generated through PSC transcriptional programming shared a higher percentage of their transcriptomic profiles with mouse embryo spMNs than spMNs produced following conventional guide differentiation protocols [84]. A potential strategy to capitalize on the faster spMN formation achieved by TF overexpression without disregarding the molecular cues of embryogenesis could be the combination of both approaches [85,86]. 

### 2.3. Breaking New Grounds in Spinal Cord Organoid Culture: Evolution from Neurospheres to Early SCO Protocols

In a pioneering study, Meinhardt and colleagues demonstrated the formation of neural tube-like structures embedding mESCs suspensions directly into an exogenous extracellular matrix (Matrigel), which generated NESTIN+ neuroepithelial spheroids. Notably, these neuroepithelial EBs displayed a homogeneous, anterior axial identity of GBX2 and HOXA2 [87], characterized by the expression of the hindbrain markers OTX2 and could be ventralized upon treatment with SAG, which induced the formation of SHH+ FP-like structures. Simultaneous treatment with RA guided dorsal–ventral patterning and posteriorized 90% of the neural cysts, which showed cervical *HOXC4* gene expression and a spatial arrangement of ISL1 + MNs, ventral and dorsal interneurons along the pseudo-DV axis, positioned in relation to the FP-like structure. A more precise recapitulation of the SpC DV domains was later recreated in hiPSC-derived spheres by dual SMAD inhibition with SB/LDN, followed by treatment with BMP4 or SAG to induce dorsalization of spinal progenitors or to trigger a developmental shift toward ventral fates, respectively, in combination with RA [88]. A similar approach generated SCOs mainly composed of dorsal neural progenitors and sensory neurons, where the heterogeneity of dorsal cell types was influenced by adjustments in BMP4 concentration and exposure duration [89]. In this study, the dorsal neurons were positioned peripherally relative to the ventral subtypes. Afterward, Hor and colleagues expanded on spMN differentiation protocols established by Wichterle et al. 2002 and Chambers et al. 2008, by embedding hiPSC-derived neural spheres in Matrigel and adapting previously published strategies for cortical organoid culture to generate SCOs [90]. They further refined the differentiation protocols by introducing patterning modifications, such as concurrent treatment with the WNT signaling agonist CHIR99021 to enhance the differentiation outcome. Their results demonstrated that the SCOs reproduced the emergence of ventral SpC NKX6.1+ and OLIG2+ progenitor populations and CHX10+ ventral interneurons. Interestingly, the organoids contained ventricle-like structures with apical-to-basal polarity, with a layer of proliferative SOX1+ cells marking the apical region, while ventral progenitors and ISL1 + spMNs were located basally. One may hypothesize whether this unique organization could be attributed to the activation of the WNT signaling pathway, known to play a crucial role in establishing apical-to-basal polarity during vertebrate CNS embryogenesis [91]. When examining the diversity of neurons along the RC axis, Hor et al. elaborated that in their organoid culture, cells corresponding to the thoracic HOXC8+ cluster of neurons were generated, whereas the same guided differentiation protocol in 2D culture only resulted in cervical identity HOXB4-expressing cells [90]. This suggests that embedding the SpC spheres in Matrigel potentially promotes the self-assembly and lineage specification of caudal progenitor cells. This hypothesis is supported by the findings of Veenvliet et al., who demonstrated that mESCs can generate highly organized “trunk-like structures” comprising the neural tube and somites when they were aggregated and embedded in Matrigel [92].

### 2.4. Increasing Complexity and Heterogeneity: Generation of SCOs from Axial Progenitors

spMNs exhibit a preferential degeneration pattern along the SpC RC axis in neurodegenerative diseases such as SMA or ALS [93]. In ALS, spMNs of the lower SpC segments are preferentially affected and degenerate first, then degeneration of spMNs progresses to the more rostral segments and also MNs of the motor cortex [94]. In contrast, certain MNs remain unaffected until the late stages of the disease, such as the oculomotor MNs that regulate the contraction of the eye muscles. A similar selective degenerative phenomenon has been reported in SMA [95]. Challengingly, the exact molecular determinants for this selective vulnerability are still unclear. Thus, generating hPSC-derived pMNs in vitro with specific RC identities may aid to elucidate the molecular underpinnings of these MNDs and discover new therapeutic possibilities. 

**Figure 2:**
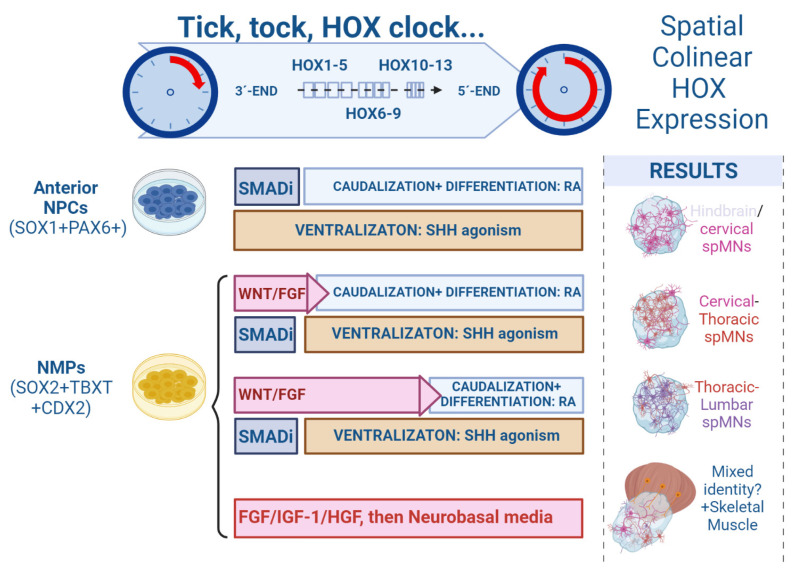
Mimicking the HOX clock to modulate axial elongation in vitro. Most protocols to form spinal cord organoids pattern hiPSCs directly into anterior neural progenitor cells (NPCs), inducing differentiation by RA and SHH pathway activation, and thus, ventral spinal cord fate and formation of hindbrain/cervical spinal motor neurons (spMNs) are achieved. In contrast, the generation of axial progenitors (NMPs) allows for the prolonged activation of the HOX clock. Hereby, the length of exposure to WNT/FGF before RA determines which *HOX* genes are expressed; therefore, different organoid protocols are designed in a way to give rise to the spMNs of a certain axial identity in a controlled manner. Prolonged maintenance of NMPs in neurobasal media seems to enable the formation of neuromuscular junction organoids, which contain spMNs of mixed axial identity that innervate the skeletal muscle part of the organoid. Created with BioRender.com (accessed on 2 May 2023).

During vertebrate embryogenesis, colinear expression of *HOX* genes in the hindbrain and SpC plays a crucial role in diversifying and assigning neural phenotypes to specific anatomical domains along the RC axis [60]. If the underlying mechanisms that regulated the pacing of the HOX clock were uncovered and replicated in vitro, it would be possible to achieve a sequential expression of progressively more caudal *HOX* gene clusters, thus generating spMNs with a specific axial fate [48,96]. It has been proven that the HOX clock can be adjusted in vitro. For instance, Mazzoni et al. revealed that stimulation of WNT/FGF signaling induced a saltatory activation of the HOX clock in mouse PSCs undergoing neural differentiation, resulting in the generation of HOXC6+ cervical and HOXD9+ thoracic spMNs [96] (Figure 2). 

The rationale followed by most protocols for achieving spMN differentiation is based on the activation–transformation model, whereby PSCs are first patterned toward neuroectoderm and afterward treated with RA or other caudalizing molecules to force them to commit to a hindbrain/SpC fate. However, recent evidence suggests that a different rationale has to be adopted to induce the acquisition of thoracic–lumbar spMN identity. Such a “primary regionalization model”, proposed by Metzis and colleagues, highlights that progenitor cells need to acquire an axial identity prior to committing to a neural fate [19,63]. It further proposes that, to achieve caudal SpC fate, it might be necessary to accelerate the HOX clock, generate the tail bud region axial progenitors, NMPs, and then push them to differentiate them into spMNs. Since past studies in mESC-derived NMPs observed a transition from neuroectoderm to mesoderm after only a few days in vitro, the main challenge of modulating the HOX clock in NMPs is to sustain their state long enough to gradually manipulate their axial identity without altering their developmental lineage toward the skeletal muscle [10]. 

Numerous studies have accomplished the generation of hindbrain/cervical or thoracic PSC-derived spMNs, following adherent or EB/SCO cultures. These protocols mainly applied the activation–transformation model of treating PSCs with TGFß/BMP inhibitors, followed by SHH-mediated ventralization and RA-induced caudalization. In contrast, only a limited number of studies focused on refining guided differentiation protocols to generate lumbar spMNs from NMPs, which can be achieved by firstly stimulating the HOX clock to express caudal *HOX* gene clusters and then inducing NPC specification. Lippmann and colleagues developed an optimized approach culturing with WNT agonist CHIR99021, FGF-8 and GDF11, followed by a stimulation of RA signaling [48]. The addition of GDF11, a TGF-β family member expressed during the late stages of axial progenitor propagation in vivo, was found to be fundamental for activating the expression of lumbosacral *HOX* genes and therefore promoting pMNs caudalization [97]. They observed an effective conversion of SOX2+; TBXT + NMPs into a highly pure neuroepithelial culture within 4 days, and an emergence of ISL1+; SMI32 + spMNs. Intriguingly, when hiPSC-derived NMPs were exposed to more than 72 h of WNT/FGF8 signaling activation before RA treatment, the expression of caudal *HOX* genes was more progressed and a stark reduction in cervical/hindbrain allocated *HOXB4* expression was observed [48]. Concluding from their findings and the previous literature, Lippman and colleagues hypothesized that the axial patterning mechanisms of caudalizing hiPSCs and NMPs in vitro may involve a temporal, biphasic mechanism in which WNT/FGF primarily control HOX gene activation, and RA controls its termination. Similarly, by culturing SpC spheroids with high concentrations of FGF2, GDF11 and RA to promote HOX clock progression, Mouilleau and colleagues observed up to 20% of lumbar spMNs (HOXC9+; HOXC10+; ISL1+). In this study, the authors concluded that the emergence of caudal spMNs along the RC axis was regulated by the timing of RA administration rather than by the duration of RA exposure. Extending the duration of WNT/FGF activation further to 5–7 days, along with TGFß inhibition to prevent BMP dorsalizing signals, researchers have been able to generate 70% of thoracic–lumbar, ISL1 + spMNs in SCO [98]. Lastly, starting the guided differentiation protocols from the enriched populations of OLIG2 + pMNs could notably reduce the culture time needed to generate lumbar spMNs. As demonstrated by Xu and colleagues, before undergoing 3D differentiation, the hiPSC-derived NMPs could be caudalized in vitro and differentiated into OLIG2+ cells that mainly expressed the thoracic–lumbar genes *HOXC9* and *HOXD11*, and not the cervical *HOXC6*. After 6 days of exposure to the NOTCH pathway inhibitor Compound E, caudal pMN progenitors derived from NMPs fully differentiated to HB9+; ISL1 + spMNs that retained their caudal identity [99] (Figure 2). To conclude, NMPs readily generate thoracic and lumbar spMNs, but additional studies are required to elucidate in which way NMPs contribute to the formation of cervical SpC and how to improve their capacity to generate cervical spMNs in vitro. 

Building on the NMPs’ potential to generate and elongate caudal SpC and the adjacent paraxial mesoderm, Gouti’s group recently developed the first neuromuscular organoid (NMO) protocol, composed by SpC-like tissue and skeletal muscle in approximately even proportions [100]. After being exposed to WNT and FGF2 for 3 days, they showed that hPSCs are permissive for efficiently making SOX2+; TBXT + NMPs that were also positive for the posterior determinant homeobox protein CDX2. Subsequent culture of the organoids in basic neural induction media, supplemented with only hepatocyte growth factor (HGF) and insulin-like growth factor-1 (IGF-1), resulted in the generation of HOXC9+; HOXC10+ trunk organoids that contained both spMNs and mature skeletal muscle. scRNAseq and immunostaining confirmed the dual lineage nature of the organoids and revealed that, while 75% of the cells expressed the neurofilament marker SMI32, only ~6% had a spMN identity. Furthermore, neuroectodermal cells also gave rise to trunk neural crest derivatives, Schwann and glia cells, whose numbers increased during the organoid culture, consistent with in vivo development. Unlike the SCO protocols described before, while focused on achieving a stratification of NMPs or NPCs to generate spMN diversity, the trunk organoids by Faustino-Martins and colleagues contained two different tissue within the same organoid, connected by functional NMJs. This represents an interesting new angle to model SpC development and disease. An alternative approach to generate an organoid system even more complex than the NMOs was proposed by Pasca’s lab, who assembled cortical, spinal and muscle organoids together after generating them separately [79]. These assembloids constitute yet the most comprehensive in vitro model of the human corticomotor circuit.

In conclusion, in the last 5 years, an impressive advance in SCO modeling has been made. There are still, however, unanswered questions regarding the optimal selection, concentration, timing and combination of signaling molecules for the optimal differentiation of PSCs into SpC-like tissue. Efforts to develop protocols for patterning hiPSCs into organoids that contain spMNs with mixed axial identities have encountered various challenges. Reduced yields of spMNs, a persistence of immature progenitors, strong rostral or caudal regional identity bias, or loss of entire spMN subpopulations are common examples. This is partially due to the lack of systematic studies on how mechanistically each morphogenetic signal contributes to the patterning of each type of neural progenitor and their specification into different spMN subtypes. Further research on this end will surely propel the generation of the SCO that best resembles human physiology.

### 2.5. Mimicking Spinal Cord Axial Elongation in SCOs

One could argue that an ideal human SpC in vitro model should be generated by the expansion of NMPs and their progressive differentiation into neural progenitors with different rostro-caudal segmentation commitment that additionally undergoes periodic elongation. Recent studies have discovered that generating gastruloids from hPSCs constitutes an effective in vitro model for studying axial specification during the earliest stages of post-implantation embryonic development [101,102]. These structures exhibit a progressive RC pattern, initiated by WNT signaling, which breaks the axial symmetry and leads to the formation of NMPs close to a signaling center similar to that of a tail bud. As time progresses, these round spheres develop into “tubular structures with singular axial extensions”, which represent a remarkable progress in simulating the morphogenetic dynamics of the developing SpC [102]. The elongated organoids mainly comprise neuroepithelial compartments, NPCs and NMPs, the number of which increased in the elongating organoids proportionally to the concentration of WNT agonist supplemented to the medium. In addition, the same study examined the expression of *HOX* genes in organoids grown with and without WNT signaling activation. Organoids cultured without WNT induction expressed anterior *HOX* transcripts and adopted a hindbrain identity, while WNT activation caused the spheroids to express hindbrain and cervical *HOX* transcripts. Interestingly, thoracic *HOX* genes started to emerge as the gastruloids were kept longer in culture, mimicking the progression of axial elongation during embryonic development in vivo. While N-cadherin and β III-tubulin expression was observed in the organoids, indicating the emergence of maturing populations of neurons, the (motor) neuronal character of the gastruloids was not further characterized. In summary, gastruloids in vitro constitute highly promising assets to study the mechanisms of neural tube formation and elongation, a process that has been partially recapitulated in murine cultures [92,101], and is only recently starting to be studied using human organoid models. Another interesting example with progressive elongation was recently established by Ebisuya’s group, in which they generated Matrigel embedded “Somitoids” comprising somites sequentially generated from hiPSCs in accordance with the segmentation clock by inhibition of BMP and the simultaneous stimulation of WNT and FGF signaling [103]. By using different concentrations of a WNT agonist, they also demonstrated how WNT signaling heavily impacts the lineage choices of NMPs between producing mesodermal somites or the neural tube with spinal progenitors. Based on this observation, a 3D culture that can create gradients of WNT agonists in microscales might enable the simultaneous generation of somites and neural tube potentially replicating the anterior–posterior complexity of the developing SpC while the organoid elongates (Table 1). 

## 3. 3D Models to Study Developmental Neuromuscular Disorders

Ethical and technical reasons confined research on developmental biology to various animal models such as chicken, frog, zebrafish and mice. Although they are valuable systems that have contributed immensely to discovering conserved principles of SpC development with ease of observation and manipulation, TFs and epigenetic regulators can function differently in human development. Moreover, the human genome contains many types of non-coding regulatory elements that could be essential for human embryos and structures to develop [109]. For these reasons, we should challenge the knowledge obtained from animal models in human-derived systems and further look for human-specific developmental mechanisms to better understand human biology and pathology. For this purpose, a suitable model is hPSC-derived 3D cultures that self-organize in cell aggregates and can undergo developmental stages to generate the pseudo-organ of interest. In the last decade, we have witnessed a drastic rise in the number of studies developing various types of brain organoids, being frequently used to uncover mechanisms of cell specification, migration, circuitry integration, structure complexity acquisition and the maintenance and evolution of the human neocortex, among others [110,111]. These organoids have been found to undergo developmental stages mimicking human development and have even provided some answers on the mechanisms underlying the increased size of the human cortex compared to non-human primates [112] and human-specific genes regulating neocortex development [113]. Organoids resembling the developing SpC have been significantly less pursued than cortical organoids and are still largely improving to become more accurate and robust models due to previously mentioned complexities governing SpC development. Human cell atlases of the developing and adult SpC obtained from single-cell sequencing approaches indicate the presence of human-specific cell types and different relative abundances of cell types with respect to mouse SpC [34,114,115]. In vitro SCO models constitute a valuable, simplified and tunable asset to address the fascinating question of how such human-specific features arise during development. 

Among these models, a very interesting one described previously was recently developed by Gouti’s group, neuromuscular organoids (NMOs) [100]. NMOs were found to self-organize into spatially segregated SpC and skeletal muscle tissues, form functional NMJs and undergo spontaneous contractions providing a valuable model to investigate the formation of the human neuromuscular system. The value of the NMOs to model neuromuscular diseases was demonstrated by treating healthy NMOs with myasthenia gravis (MG)-causing autoimmune antibodies derived from MG patients, which resulted in a prominent decrease in muscle contractions, characteristic of MG pathology. Another recently reported 3D system to study human SpC development was engineered by Pasca’s group and consisted of more complex assembloids, composed of cortical, SpC and skeletal muscle organoids joined after the generation of each of the three components separately [79]. The authors demonstrated that the three parts of the assembloids were functionally connected, and that cortical neuron stimulation resulted in muscle contraction, proving the establishment of at least two functional synaptic contact, thus providing a remarkably useful model to study the development of corticomotor circuits in physiological and pathological conditions. 

An organoid system highly promising for studying the earliest phases of SpC formation and the molecular basis of developmental abnormalities in vitro was developed by Martinez-Arias’ group using mouse ESCs. These 3D aggregates, termed gastruloids, contained the three germ layers, underwent symmetry breaking and were able to elongate in an axis resembling gastrulation in vitro [116,117]. In a later study by Veenvliet et al., the authors generated more advanced gastruloids by embedding these mouse SC aggregates into Matrigel, which resulted in the stimulation of complex morphogenesis and the formation of somites and neural tubes in trunk-like structures with elongated shapes similar to the tail bud of a developing embryo [92]. When cultured for 5 days, these organoids were found to contain NMPs, somites and committed SpC progenitors in addition to notochord-like cells. The single-cell transcriptomic characterization of these trunk-like structures showed embryo-like cell differentiation dynamics and a high complexity of cell states matching mouse E7.5-E8.5 embryos, making this model highly effective for studying the earliest phases of SpC tissue morphogenesis and the potential interactions of spinal progenitors with somites and endothelial progenitors [92]. Intriguingly, human ESC-derived gastruloids exhibit a shorter elongation in the AP axis than the mouse gastruloids [101] and the morphogenesis of neural tube and somite-like structures has not been reported so far. These findings suggest that these gastruloids require further optimization to more accurately recapitulate the earliest phases of human embryo development. Additional research building on the knowledge acquired from murine gastruloid models will likely speed up this process (Table 2).

## 4. 3D Models to Study Neurodegenerative Neuromuscular Diseases

In 2019, around 300,000 people worldwide were diagnosed with a MND [123]. Motor neuron diseases (MNDs) are a heterogeneous group of disorders characterized by the progressive degeneration and loss of the cortical and/or spMNs. The two most common MNDs are ALS, which is mainly sporadic and late-onset, and SMA, predominantly genetic and early onset. More rare MNDs include progressive bulbar palsy, primary lateral sclerosis and progressive muscular atrophy. The molecular mechanisms underlying MNDs are not well understood and therefore the need to develop successful treatments persists. A major obstacle to fully uncover the biology of these diseases is precisely the out-of-reach location of the MNs, which makes longitudinal in vivo studies extremely complicated. Using in vitro cultures from patient-derived cells has proven to be a valuable approach to tackle this challenge. When compared to MNs derived from healthy hiPSCs, patient-derived MN studies can shed light on molecular mechanisms causing degeneration. These in vitro methods can also be used as powerful platforms for drug testing that may improve the reliability of preclinical trials and decrease dependence on animal experimentation. Moreover, by utilizing differentiation protocols that generate MNs via guiding hiPSCs specification through neural progenitor cell fates, neurodegenerative diseases (NDs) with potentially developmental underlying pathologies can be studied with no ethical hurdles, as has been recently reported for Huntington’s disease [124] and SMA [90,108]. Additionally, MN co-cultures with other cell types, such as skeletal muscle and microglia, further enable the study of the muscular and inflammatory aspects of MNDs, with a reported critical contribution to disease progression or even initiation [125,126,127].

### 4.1. Amyotrophic Lateral Sclerosis (ALS)

Approximately 10% of ALS cases are familial (fALS) while 90% are sporadic (sALS) [127,128]. So far, more than 30 genes have been linked to ALS pathology and are involved in a large plethora of cellular processes [128,129]. Among these genes, the most extensively studied ones are *SOD1*, *TARDBP*, *FUS* and *C9orf72.* Several impaired pathways such as RNA metabolism, vesicle trafficking, cytoskeletal dynamics and axonal transport as well as the accumulation of misfolded protein aggregates have been reported being contributors to the disease. However, the precise order of events contributing to neuronal death remains elusive. Extensive research on these four genes, and many others, is ongoing worldwide to elucidate common and mutation-specific altered pathways that could uncover the disease origin, the contribution of each of them to the initiation and progression of the pathology and the interplay between MN intrinsic and cell non-autonomous alterations [128,129]. A valuable tool for ALS research in recent decades has been MNs generated from patient-derived iPSCs as they recapitulate some of the pathological changes observed in post-mortem SpCs. Most studies using human in vitro models to study ALS have followed differentiation protocols on hiPSCs grown in monolayers or aggregated as EBs and patterned toward spMN fate that are later dissociated and studied as cultures of relatively homogeneous neuronal populations [130]. In addition to these MN-based studies, reports using different types of brain or SpC-like organoids composed by a diverse range of cell types of the neuromuscular system (including muscle cells, interneurons, astrocytes, microglia and sensory neurons) to model ALS are also emerging [105]. 

Eggan’s group was the first to generate hiPSCs from a patient carrying a familial *SOD1* mutation and differentiate these into MNs [131]. As more patient-derived iPSC lines were established, numerous studies have reported the cellular impact of ALS-linked mutations such as: altered RNA metabolism, mitochondrial dysfunction, neurofilament aggregation, altered signaling pathways, MN hyper- and hypo-excitability, altered stress granule dynamics, impaired lysosomal biogenesis and the autophagy and dysfunction of nucleocytoplasmic transport [128]. Large-scale studies conducted on hiPSCs derived from patients affected by fALS and sALS aimed to identify disease-relevant common pathways and to uncover novel players are also emerging. A prominent example of this is a recent study that generated hiPSCs-derived MNs, employing a 2D protocol, from 341 ALS patients and 92 healthy individuals, establishing a highly reliable platform for disease modeling and drug discovery [132]. This large cohort of hiPSC lines enabled the identification of sex-related differences in disease phenotypes, such as a higher percentage of MNs being produced by the male lines. Even though these studies are providing fascinating insights into the complexity of ALS pathologies, both MN cultures and SCO-based models have been reported to have immature transcriptomic and electrophysiological profiles [133], in part due to the abolishment of aging-associated epigenetic marks during somatic cell reprogramming [134,135]. This could constitute an important limitation, especially when used to model a late-onset ND such as ALS. An approach to circumvent this issue is the direct lineage conversation of patient somatic cells into neurons by overexpressing neuronal commitment TFs in patient donor cells [136]. MNs converted from fibroblasts from old donors conserved aging-related transcriptomic signatures and displayed nucleocytoplasmics defects associated with aging [137]. This strategy has been applied to study ALS. For example, *LHX3*, *ISL1*, *NGN2* and *SOX11* overexpression in *FUS*-mutated fibroblasts generated lineage-converted MNs that showed soma shrinkage, hypoactivity and inability to form NMJs [138]. 

Even though the use of patient-derived iPSCs for ALS research has exponentially grown in the last decade, only a few studies have utilized complex 3D-based differentiation protocols. As described above, using more physiologically relevant, tunable, and tractable 3D models should not only recapitulate more closely the human disease but also uncover disease mechanisms that might remain otherwise masked in more simplified 2D models. A recent study by Pereira et al. established sensorimotor organoids that contained microglia, endothelial cells and skeletal muscle fibers in addition to MNs by generating NMPs from hiPSCs and allowing them to differentiate into mesodermal and neuronal lineages [105]. The authors observed a decreased muscle contraction frequency when the sensorimotor organoids were generated from hiPSCs carrying mutations in *C9orf72* or *FUS* with respect to organoids generated from healthy individuals, supporting a NMJ dysfunction in ALS. To conduct a detailed analysis of NMJ structure and composition, they switched to isogenic hiPSC lines engineered by introducing *SOD1*, *TARDBP* or *PFN1* mutations into a healthy control line. Experiments revealed a reduced number of NMJs in the SOD1 and PFN1 mutant organoids and defective innervation in the established NMJs in TDP43 mutant organoids. Since no changes were found in axonal growth or skeletal muscle area across the different genotypes, they concluded that the ALS mutations lead to a structural inability to form NMJs that needs to be explored further to determine if this disease phenotype has a degenerative or developmental nature [105]. Importantly, they also showed that the inter-organoid variance in cellular composition was markedly decreased in the ones generated from the isogenic cohort compared to the ones derived from different donor hiPSCs. These findings highlight the importance of isogeneity for functional comparisons and replicability.

The next generation 3D approach to model MNDs is organ-on-chip, in which different cell types can be cultured in separate compartments by combining the use of hydrogels and microfluidic devices. This strategy guides cells to ensemble in 3D structures while precisely controlling their microenvironment (biological cues, nutrient availability, air composition and pH) to better simulate human physiological conditions [139]. An interesting study based on this micro-3D approach generated motor units containing optically excitable MNs and muscle connected through functional NMJs [119]. When these motor units were derived from ALS hiPSCs, reduced muscle contractility, an apoptosis of the muscle fibers and MNs degeneration were observed, which could be rescued by Rapamycin treatment [120], demonstrating the power of these “nerve organoids” as useful models to study the contribution of NMJ pathology to MND progression (Table 2).

### 4.2. Spinal Muscular Atrophy 

SMA is an autosomal recessive neuromuscular disease and the leading genetic cause of infant death. It is caused by mutations or a full deletion of the *SMN1* gene, which codes for survival of motor neuron (SMN) protein. The complete lack of SMN is incompatible with life, and homozygous deletion of *Smn* in mice results in death at the gastrulate stage [140]. A paralog gene, *SMN2*, only present in humans, allows patient survival and partially compensates for *SMN1* loss. Importantly, a translationally silent point mutation in *SMN2* exon 7 results in the skipping of this exon in most of the *SMN2* transcripts during mRNA splicing, resulting in a truncated and unstable form of the protein. Approximately 10% of the *SMN2* transcripts retain exon 7, and thus code for the full-length functional SMN protein [141,142]. The number of *SMN2* gene copies varies across individuals (zero to six copies) and determines the onset and severity of the disease (type 0–4). Higher *SMN2* copy numbers translate into higher levels of full-length SMN and a milder course of the disease and a longer life expectancy, while lower copy numbers result in severe forms and death, often occurring during infancy [143]. SMN is an ubiquitous protein and has essential housekeeping functions, such as mRNA splicing, RNA metabolism and axonal mRNA and vesicle transport [144].

To investigate the spMN degeneration caused by SMN deficiency scientists initially relied on murine models, generated by introducing a different version of the human *SMN2* gene [145,146] while knocking out the mouse *Smn*. These in vivo SMA models replicate major disease hallmarks such as spMN loss, NMJ degeneration, muscle wasting and a shortened lifespan [147]. These accurate models have remarkably contributed to the development of three FDA-approved therapies designed to increase full-length SMN abundance in patients by promoting the inclusion of *SMN2* exon 7 or by an AAV-mediated *SMN1* gene [148]. Despite all these advancements, current therapies are not curative and a wide variability in the patients’ response to the therapies has been reported [143]. Additionally, fundamental questions on disease mechanisms remain to be solved, such as how the deficiency of a protein with such fundamental functions in all cell types results primarily in the death of spMN. The use of patient-derived iPSCs to model SMA have become prevalent in recent years; however, most of the studies were conducted in 2D MN cultures or using EB-based differentiation protocols to generate monolayers of spMNs used in 2D experimental assays. SCOs as tools to further explore SMA biology and the search for additional therapeutic candidates are only beginning to arise. 

A pioneering hiPSC-based study on SMA was conducted by Ebert and colleagues, where they reprogrammed fibroblasts from a patient affected by a severe SMA form and from his healthy mother into hiPSCs and differentiated them into MNs using a EB-based method [149]. Soon, human-based in vitro studies will start to uncover differences with previously published model organism-based reports, raising some controversy that only helped to move the field forward. For example, while evidence from drosophila and mice models suggested that a sensory neuron induced the loss of MNs [150], co-culture experiments of sensory neurons and spMNs derived from SMA hiPSCs showed that MNs death was found to be independent of sensory neurons [151]. Searching for the pathological mechanisms of the disease, Makhortova and colleagues performed the first large-scale small molecule screen on EB-dissociated spMN cultures derived from patient SMA hiPSCs, and found inhibitors of PI3K/AKT/GSK-3 signaling cascade as a potential therapeutic agent [152]. The muscle component of this disease has also been studied using hiPSC-derived cells. Patient-derived MNs co-cultured with murine muscle cell lines enabled the discovery of defective NMJ formation prior to the death of spMNs, which suggested that SMA spMNs are not successful at establishing NMJs, as it was reported in ALS [153]. Studies using a 2D-based differentiation of hiPSCs found that SMA spMNs are hyperexcitable [154] and present an aberrant axonal mitochondrial transport [155] as key contributors to their degeneration. Rubin’s team following EB-based spMN differentiation protocols found that the endoplasmic reticulum was affected by SMN depletion due to the accumulation of misfolded proteins, which triggered an unfolded protein response and ER stress pathways [156]. Interestingly, they further observed that SMN protein levels across spMN with the same genetic background (derived from healthy individuals or from patients affected by different SMA severities or fALS forms) were highly heterogeneous [157] and that such heterogeneity directly correlated with the spMN probability of survival, with the low-SMN expressing MNs being more vulnerable to different stressors. This report highlighted the significance of utilizing single-cell approaches to unravel relevant disease mechanisms [157]. A different study by the same group also discovered that SMN protein is degraded by p62-mediated selective autophagy, and not only by the ubiquitin/proteasome system as commonly believed, offering therefore an additional route to increase SMN protein levels [158]. Although most hiPSC-based SMA studies have focused on the progressive degenerative nature of the disease, some have started to evince the potential contribution of an altered development. This angle can be readily undertaken by using 3D differentiation protocols that recapitulate the embryological development of the neuromuscular system. In the first study that used SCOs to model SMA, alterations in MNs biogenesis was not observed [90]. However, by generating the first CRISPR/Cas9-mediated isogenic cohort of SMA corrected hiPSC lines, and following a physiologically relevant SCO model that our group has developed, we recently discovered that markers of NSCs, early pMN and spMNs are significantly reduced and expressed in a temporally abnormal fashion [108]. In particular, a marked decrease in the expression levels of *SOX2* and *NESTIN* brought forth developmental alterations in NSCs/NPCs as potential initial drivers of SMA pathology [108] (Table 2). While organoid-based studies for SMA are still very few, future research will likely benefit from employing these models to dissect the developmental vs. post-mitotic contributors of premature MN degeneration in SMA and to systematically characterize the cell-autonomous and non-autonomous mechanisms of disease, their order of appearance and the crosstalk between them. 

## 5. Stem Cell Types and SCOs for Cell Therapy in Motor Neuron Diseases 

Except for SMA, there are very few therapies available to treat MNDs. While fundamental research is heavily ongoing to identify new pathways and molecular targets that could open unexplored treatment avenues, stem cell-based therapy is emerging as a potential strategy to replace lost spMNs and/or to protect the remaining neuromuscular units. Numerous preclinical and some clinical studies have revealed that therapy with different types of SCs could represent a promising strategy to slow down the muscle denervation and MN degeneration in neuromuscular disorders. The main sources of stem cells used in clinical trials are mesenchymal SCs (MSCs, obtained from either the amniotic membrane or adult bone marrow or adipose tissue), hematopoietic stem cells (HSCs, obtained from umbilical cord, bone marrow or peripheral blood) and NSCs (obtained from fetal brain or derived from ESCs). ALS has been the MNDs for which the highest number of cell therapy-based clinical trials have been approved in the past two decades to test for safety, tolerability and early efficacy. Although the vast majority of the completed trials have proven safe, regardless of the type and number of cells transplanted and the delivery route, the functional improvement of the patients has been modest, with a mild reduction in the slope decline or diminished levels of pro-inflammatory cytokines being the most common positive outcomes [159,160]. Most approaches used autologous bone marrow-MSC [161], known to secrete neurotrophic factors and promote cytoprotection, sometimes in combination with already approved drugs (e.g., Riluzole), and administered via intramuscular or intrathecal injections or a combination of both. The slow disease progression for a short time window after the transplantation of fetal-SpC-derived NSCs has been also reported [159] and several clinical trials using genetically modified PSC-derived NPCs are currently ongoing [159,160]. Interestingly, the first FDA-approved one showed that, in postmortem samples of the ALS patients who died of disease progression, the SpC transplanted NPCs engineered to produce GDNF were still engaged in the tissue almost 2 years post-transplantation [162]. These encouraging results indicate that NPCs can engraft, survive and remain functional for long terms when transplanted in the adult human nervous system.

In ALS animal models, besides BM-MSC, HSCs and ESC-derived NSCs, hiPSC-derived NPCs have been delivered systemically and via intracerebroventricular, intrathecal or intra-SpC parenchyma injection with different efficacy outcomes. Most studies have reported some beneficial effects, including: delayed onset or disease progression, temporarily improved motor function, reduced reactive gliosis or inflammation, and in some cases, preserved NMJ, MN survival and prolonged lifespan, irrespective of the cell-type source, the number of cells transplanted or the delivery route and number of interventions [159,160]. The positive outcomes have been associated with a plethora of effects, including: immunomodulation and anti-inflammatory properties of the transplanted cells [163], neurotrophic factor release [164], decrease oxidative stress [165], activation of pro-survival pathways [166] or repair of damaged blood SpC barrier [167]. These promising findings were observed despite the reported limited migration of the transplanted cells into the SpC parenchyma and their reduced differentiation into neural cells (even when NSCs/NPCs were the cell source [168]), which supports a main cytoprotective paracrine role of the transplanted cells and encourages further preclinical research to improve this current hurdle. 

Despite the tumorigenicity potential of hiPSCs if the cells or organoids derived from them are not fully differentiated, using hiPSCs as the cell source offers the critical clinical advantage of no immune rejection with the possibility of autologous cell transplant. With this scope in mind, numerous preclinical studies have reported survival, functional integration, axonal elongation and muscle preservation after hiPSC-derived NSCs/NPCs transplantation in ALS [159] and SMA [169] animal models. Generally, it seems that the beneficial effect relates to the engraftment’s span in the host tissue, the time of intervention and the ability of the cells to differentiate into neuronal or glial cells. Additionally, better outcomes have been achieved when the iPSC-derived cells are modified to express neurotrophic factors and/or modulate the immune response. In 2012, Corti and colleagues showed that the transplantation of SMA hiPSC-derived MNs, previously corrected in vitro with single-stranded oligonucleotides to convert a *SMN2* into a *SMN1*-like gene, into the SpC of newborn SMA mice modestly ameliorated disease phenotype and increased the mouse lifespan [170]. The rapid and successful development of gene and molecular therapies for SMA has slowed down preclinical cell-replacement-based studies as an alternative therapy for this disease. Nevertheless, the possibility of using cell therapy in combination with SMN-targeted approaches as a source of trophic factors to enhance therapeutic outcomes remains open.

Recently, hiPSC-derived organoids as entire entities have arisen as another potential source for transplantation, providing advantages over former approaches using the suspension of dissociated cells. Daviaud et al. showed that hiPSC-derived cerebral organoids transplanted into an injured mouse cortex survived better and displayed a more robust host vascularization, resulting in a better engrafting than transplanted NPCs differentiated from the same iPSC lines [171]. The authors reasoned that this could be due to the larger pool and differentiation stages of NPCs present in the organoids that might improve engagement with the host tissue. Although the integration of a functional vascular network in brain organoids needs to be further developed, and is still to be implemented in SCO models, it has been shown that human brain organoids transplanted in adult mouse brain do get vascularized by the host vasculature network [171,172]. These studies opened the interesting possibility of combining human neural organoid with in vivo transplantation into the nervous system of control or ND animal models to improve disease modelling and testing of therapeutic candidates under the best yet physiological conditions. Intense investigations are ongoing to establish the best brain organoid cellular composition and their developmental stages to ensure the functional engraftment of the replaced cells. For instance, Kitahara and colleagues showed that while six-week old human ESC-derived cerebral organoids were more efficient at extending axons in transplanted mouse cerebral cortices compared to ten-week old organoids, they also overgrew due to their high proliferative cell content [173]. Studies such as this highlight that the precise characterization and identification of the right proliferative/differentiation state of the cells to be transplanted constitutes an essential factor to overcome for cell therapy to become a reality to treat neurological disorders.

The transplantation of hiPSC-derived brain organoids has recently been proven valuable to reveal neuronal connectivity phenotypes in rare neurological disorders that were masked in the limited maturation stage of the current in vitro models. By transplanting genetically engineered human cortical organoids derived from individuals with Timothy syndrome into the somatosensory cortex of newborn rats, Pasca’s group demonstrated that the transplanted cortical neurons not only matured further and functionally engaged into the host neuronal circuitry, but also uncovered abnormal electrophysiological properties in the patient-derived neurons [174]. Structural and functional integration of human forebrain organoids into the visual cortex of adult rats post-injury has also been recently reported [175,176]. These stimulating studies are revealing that different types of human brain organoids can successfully incorporate into distinct regions of the newborn and adult nervous system of mammals and mature furthers, harmonizing with the host tissue and even replacing lost functions.

Other cells aside from NPCs or pMN differentiated from iPSCs have been transplanted into rodent models of neuromuscular disorders as an attempt to slow down disease progression, for example, muscle progenitors. Because of the MN degeneration, their muscle targets denervate and eventually deteriorate as well. Initially, efforts were placed at generating and expanding in vitro muscle stem cells (satellite cells) from hiPSCs. However, their very limited proliferation capacity has not resulted in satisfactory clinical outcomes yet. Research into generating other muscle progenitors using hiPSC-derived muscle organoids, such as mesoangioblasts and pericytes, as well as at increasing their migration potential [177] once engrafted into the host tissue are ongoing [178]. Immune cells, together with MSCs and NPCs, have been a main subject of cell therapy trials due to their immunosuppressor or immunomodulation capabilities and the impact of these in neuronal death upon acute brain damage or in NDs. M2 macrophages derived from hiPSCs from healthy controls and patients affected by sporadic ALS or carrying *C9orf72* mutations were shown to have strong immunomodulatory activity in vitro [179]. Therefore, the administration of a “cocktail” of organoid-derived cell types aimed at targeting different neuroprotective aspects (cell replacement, immunomodulation, neuroplasticity, cytoprotection) is arising as a promising strategy to be pursued in the future. It is important to keep in mind that, if hiPSC-derived organoids/cells are to be used for autologous cell therapy transplant in a patient affected by MNDs with a known genetic cause, those cells would probably need to be engineered to correct such genetic alterations first. Importantly, genome editing approaches could be employed to not only replace the cell type of interest, but to program those cells to deliver growth [180], neurotrophic factors [181], neurotransmitters [182] and/or anti-inflammatory agents. Genome editing technology can also be used to engineer iPSCs so that certain TFs are switched on or off in a cell-type and temporal-control manner to enhance progenitor differentiation after engraftment.

Although brain and SCOs and/or their derivatives hold great promise as a form of cell replacement therapy that could be used alone or in combination with other more standardized approaches, such as target-specific drugs or gene therapy, a long path ahead is expected before they are to become a real therapeutic alternative. Critical biological features of the organoids, such as cell type(s) to be transplanted, cell suspension or organized structures, proliferation/differentiation stage, number, location and delivery route, as well as the host, such as the degree of damage at transplantation, cell loss, reactive gliosis, inflammation and the functionality of neuronal circuitry, will need to be fully understood to ensure the safety and efficacy of the engraftment. 

## 6. Current Challenges and Perspectives for SCO Culture

Even though SCOs hold great promise for understanding human SpC development and MND biology, they are so far only able to rudimentarily represent the complexity of the SpC. Several studies have started to evaluate how accurately the emerging cell populations in these models reflect the in vivo counterparts [100,102]. A plethora of challenges remain, ranging from reproducibility regarding organoid differentiation outcomes, limited axial spMN heterogeneity, a lack of neuronal maturation or insufficient nutrient/oxygen supply in the organoids’ core. 

**Figure 3:**
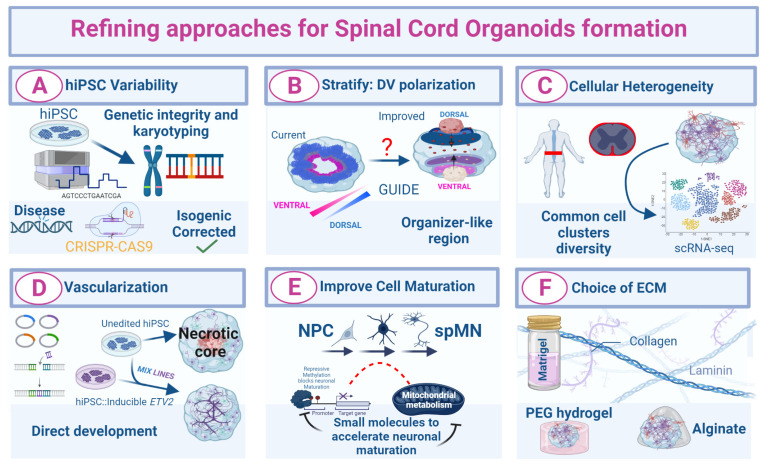
A plethora of options for the protocol optimization of spinal cord organoids (SCOs) can be explored. (**A**) Through testing the genetic integrity of hiPSC lines, researchers may find cell lines which are optimally suited for organoid modelling studies due to the low prevalence of background mutations. Moreover, disease modeling studies can be improved thoroughly by introducing isogenic lines as alternative controls. (**B**) Stratification of the SCOs along the dorsoventral (DV) axis may be achieved through protocol optimizations yielding organizer-like regions. (**C**) Moreover, adding functional vascularization can facilitate nutrient supply and prevent the formation of a necrotic core. (**D**) Benchmarking the cellular heterogeneity observed in the organoids against scRNAseq datasets from human or murine spinal cords can provide valuable input on which cellular subpopulations are contained in the in vivo organ, and how well the diversity of these cell clusters is recapitulated in an in vitro model. (**E**) The limited SCO maturation in many protocols can be improved by different small molecule-based strategies. (**F**) Matrigel, hydrogels and alginate, the most commonly used extracellular matrices for embedding spheroids or organoids. Created with BioRender.com (accessed on 2 May 2023).

As discussed previously, SCOs can be derived from hiPSC through the formation of EBs and subsequent guided differentiation. As hiPSCs/hESCs are used as the “starting point” of the SCO culture, it is fundamental to consider potential pitfalls concerning their handling and their internal properties. Differences in epigenetic marks, chromatin modifications, mRNA and protein synthesis dynamics can vary greatly between hiPSC lines generated from different donor cells or even lines generated from the same donor but by different groups [183,184]. Further, after accounting for genotype and sex, high variability in the differentiation potential among hiPSC lines has been described [185]. Several groups have reported that the application of the same differentiation protocol to different hiPSC lines, or only the successive passages of the same hiPSC line, often led to varying differentiation efficiencies [186,187]. In comparative disease modeling studies, the different genetic background of control hiPSC lines compared to disease lines can impose challenges for reproducible organoid formation and data interpretation. Additionally, the responsiveness of hiPSC lines to small molecules supplemented into the culture medium also varies, which greatly affects the outcome of the patterning protocols [49,79,102]. Furthermore, since not all organoid modeling studies are based on the hiPSC lines of isogenic background, unknown mutations in the genome of the somatic cell donor may impact the dynamics of organoid development or the appearance and degree of disease phenotypes [108] (Figure 3A). Subsequently, questions and criticism about the standardization of such organoid approaches for modeling neuromuscular development and disease need to be addressed. Experimental variability is also evident in trunk organoids and spinal gastruloids, where some recent protocols showed limited reproducibility. For instance, somite-like structures are observed in only 50% of Matrigel-embedded murine trunk-like gastruloids, and the presence of an elongated morphology as well as caudal progenitor formation varied significantly across samples, even within the same experiment [92,102]. Although the intra- and inter-experimental variabilities are a handicap to overcome in ORG-based research, guided differentiation protocols where the original PSCs are exposed to patterning molecules mimicking development have been proven to be more robust than the unguided ones, where PSCs are minimally treated at the earliest phase of the culture and later cultured only in a medium that favors neural specification [188].

Despite intense efforts to reproduce the cell-type-specific gene expression patterns observed in the SpC in vivo, we are still far from precisely replicating development in vitro. Certain marker genes that define specific cell clusters may be absent or decreased, which indicates a different ratio of distinct neural and non-neuronal cell populations, a common issue in current neural organoid models [7,188]. The presence of a large heterogeneity of cell types in organoids is especially relevant for disease modeling, as pathologies often affect more than one cell type, and such heterogeneity is important for studying non-cell autonomous disease mechanism. There are numerous examples in the in vitro and in vivo disease modeling that support this claim. In some mouse models of CNS-affecting human diseases, the disease phenotype was not fully recapitulated when a mutant gene product causative of an inherited ND is specifically expressed in known vulnerable neuronal types [189]. Similarly, the degeneration of spMNs can be induced by the selective expression of mutant proteins in surrounding non-neuronal cells such as glia or astrocytes [190,191]. In recent years, using scRNAseq several studies have characterized the cellular composition of the mouse and human fetal and adult SpC [35,36,37,115]. These atlases constitute fantastic tools for benchmarking the cellular heterogeneity and the GRNs governing cell identity specification and maintenance achieved in SCOs. While SCOs offer improved depths in capturing cellular complexity compared to regular adherent MN cultures, the absence of anatomical polarization poses pitfalls for studying complex neuronal functions [104]. This creates an additional challenge for the investigation of neuronal migration, axonal pathfinding and circuit formation during morphogenesis. While some organoid protocols indeed generated graded TF expression and demonstrated the emergence of organizer-like regions with ventral identities, others did not contain clear topographical organization and showed a sporadic localization of NPCs [79,88] (Figure 3B). The heterogeneity of cell populations and the temporal aspects of their specification can be tracked by lineage-tracing-based methods and scRNAseq [192,193,194] (Figure 3C).

While strategies aimed at reducing inter-organoid heterogeneity, such as by simply starting with a reduced number of seeded cells, have also generally improved the organoid health as a result of a smaller size, the general absence of vascularization in the SCOs poses limitations to the physiological modelling of the SpC. The neurovascular system plays a crucial role in supplying oxygen and nutrients to the brain and SpC, facilitating the growth and development of neural tissue and controlling tissue homeostasis by [195,196]. Paracrine interactions between neurons and vasculature during development are well known, as endothelial cells secrete growth factors that support the expansion and maturation of neuronal progenitors [197]. Taking this further, all currently published SCO models lack functional vascular systems. Consequently, organoids grown for extended periods can develop necrotic tissue in the innermost center of the organoid, potentially affecting the development and survival of progenitors and differentiated cells [198,199]. As previously employed in cortical organoids, one option to solve this issue of oxygen and nutrient perfusion is the sliced organotypic culture method [200,201]. However, this approach partially severs the axonal connectivity between neurons and potentially affects the accuracy of development and disease modeling. Additional recent strategies attempting to overcome the deficient organoid nourishment include incorporating endothelial cells by following different approaches. Some studies have reported a limited positive outcome by embedding endothelial cells together with brain organoids in Matrigel droplets or by transplanting endothelial cells from different precedence into the organoid directly. Although such primitive endothelial networks provide a potential platform for modeling the neurovascular niche and blood–brain barrier, they are unable to effectively facilitate nutrient delivery into organoids and prevent the formation of a necrotic core. Another reported method is the viral or transposase-mediated overexpression of TF driving the differentiation of PSCs into endothelial lineages (i.e., *ETV2*) (Figure 3D). When the genome engineering of these cells includes an inducible control of those TFs, different lines can be combined together to generate the organoids, and only upon supplementing the culture medium with the right molecule (often tetracycline and derivatives such as doxycycline) at the desired time, vascular endothelial cells are then induced to differentiate the intertwined with the neural tissue [202]. Using a similar approach, Cakir and colleagues generated cortical organoids that resembled several blood–brain barrier features, such as increased expression of tight junctions and trans-endothelial electrical resistance. As a result of the vascularization-like network, the authors found reduced TUNEL staining and HIF-1α hypoxic cells [203]. Following a different approach, Sun and colleagues successfully generated CD31+ brain vessel organoids by fusing cortical organoids to vessel organoids made from hiPSCs treated with WNT signaling activators and subsequent exposure to the growth factor VEGF. This and other recent studies [204,205] demonstrate how a robust and highly specific vascular network with functional permeability properties can be achieved in vitro and added by different means to SCOs.

Transcriptomic studies have raised questions regarding the maturity of hiPSC-derived cortical and spMNs in vitro, indicating that their global transcriptome electrophysiological profiles resembled more closely to that of fetal neurons. This constitutes a limitation for modeling late-onset MNDs such as ALS [184,206]. Proteomic profiles, cellular integration, neural circuitry establishment and functionality are other parameters that can be measured and crosschecked with in vivo studies to determine the maturation state of the SCOs. Very few SCO studies have conducted a comprehensive examination of neuronal maturity markers such as PSD95 and SYN1 [107]. It would be interesting to explore whether extending the length of the culture protocols aids the acquisition of more mature properties or whether additional external intervention (chemically or genetically based) is needed to increase the maturation rate of SCOs in vitro. A recent study by Studer’s group claimed to have found a cocktail of small molecules targeting chromatin remodeling and calcium-dependent transcription that increases the prevalence of maturity markers and synaptic transcripts in cortical organoids [207]. An interesting alternative approach to accelerate the maturation of cortical neurons was found by enhancing a mitochondrial metabolism through the inhibition of pyruvate dehydrogenase kinase [208] (Figure 3E). Applying these or similar strategies to SCOs may facilitate the conversion of remaining progenitor cells or immature neurons into spMNs with mature synaptic structures, thus improving the applicability of SCOs to model late-onset MNDs. 

In conclusion, despite the significant progress made in the last decade in optimizing guided differentiation organoid protocols to mimic the developmental cues that orchestrate SpC formation, notable challenges remain. Achieving robust SCO cultures that accurately replicate the anatomical regionalization, cytoarchitecture and cell-type specification of the SpC constitutes an ambitious task. However the continuous refinement of protocols —perhaps by applying microfluidic-based morphogen gradients and next generation functionalized matrices—as well as standardization approaches for hiPSC quality control and the same genetic background should push this endeavor. 

## 7. Future of SCO Becoming Present: Scaffolding Matrices and Microfluidic Devices

A major determinant for the success of 3D cell cultures in terms of modeling complex tissues lies in the choice of extracellular matrix (ECM) (Figure 3F). Physiologically, each tissue environment has a unique combination of collagen, elastin, proteoglycans and glycoproteins secreted by cells residing in the tissue and forming a structural network not only physically supporting cells to grow in 3D but also providing chemical and physical cues that can regulate cellular responses critical for the function of the tissue [209]. Despite being an important component, in the earliest 3D cultures, stem cell aggregates or spheres were not embedded in exogenous matrices but maintained scaffold-free. The approach to expand stem cells in a 3D format that prevails today consists of transferring the cells to low-adherence surfaces that prevents attachment and forces cells to cluster together based on random interactions [210]. This technique was improved by incorporating rotation to the culture to promote the formation of perfect spheres and an even distribution of nutrients and small molecules, which has led to today’s rotating ultra-low attachment cultures containing the suspensions of cells in plates, spinning flasks or bioreactors for larger-scale production [211]. Today, culturing stem cell spheres to generate spheroids or organoids both in the presence and absence of an exogenously added ECM is widely common. Interestingly, a recent study comparing organoids cultured with and without the predominantly used ECM, Matrigel, showed massive differences in self-organization and rate of differentiation in the early stages of the development, demonstrating the importance of ECM choice to recapitulate early development in vitro [212]. Likewise, a separate report demonstrated that gastruloids only underwent extensive morphogenesis to form neural tube flanked by somites when embedded in Matrigel [92]. Matrigel is an extract derived from Engelbreth-Holm-Swarm murine tumor identified in the late ‘70s as a type of ECM typically abundant in the basal side of the epithelium [213]. The relatively easy extraction of Matrigel and its ability to induce functional differentiation and morphogenesis [214,215,216] led to its wide application in biology. Despite its widespread use, the pursuit of other ECMs suitable for 3D cultures did not perish because Matrigel is a very complex substance containing more than 1800 proteins [217] of which the exact composition is unknown. Additionally, variability between Matrigel batches has been shown to rise due to differences in age, weight, and genetic background of the source mice [216], creating reproducibility issues. Furthermore, Matrigel’s composition cannot be tailored to the tissue of interest, which can be critical for accurate in vitro modeling given that ECM’s composition changes between different organs depending on the function and developmental dynamics of the tissue [209]. Additionally, it has been shown to be physically heterogeneous by having variable elasticity in different regions of a Matrigel droplet [218]. These reasons and the expansion of organoid-based research in the last decade propelled the development of novel ECMs with xenogenic-free, chemically defined and tunable compositions that have superior mechanical and functional properties and could lead to more physiologically relevant in vitro cultures or to the improvement of certain organoid features, such as neural maturation [219]. 

Polyethylene glycol (PEG)-based hydrogels have emerged as chemically defined, scalable and customizable alternatives, with almost half-cost and ease of scalability without batch variability that, in some cases, have proven to have an equal or superior performance in cell culture systems with respect to Matrigel [220]. However, these hydrogels need to be tailored to ensure the survival, growth and proper differentiation of each organoid type. A promising customizable ECM for SCO culture has been recently reported to be alginate [106], a natural polymer extracted from brown seaweed. Growth factors and morphogens can be added to these hydrogels to functionalize them and, hence, generate biological cues for guiding the self-organization of cells. For example, the functionalization of hydrogels with FGF2 has been shown to modulate angiogenesis in the 3D cultures of endothelial cells [221]. These hydrogels can also be loaded with patterning molecules, releasable in a controlled manner, by adjusting certain gel properties such as the percentage of heparin, the charge or the gel pH [222]. This approach can be valuable for increasing the cellular heterogeneity in the organoids by creating morphogen gradients that should also induce the polarization of the SCOs in DV and AP axes. 

**Figure 4:**
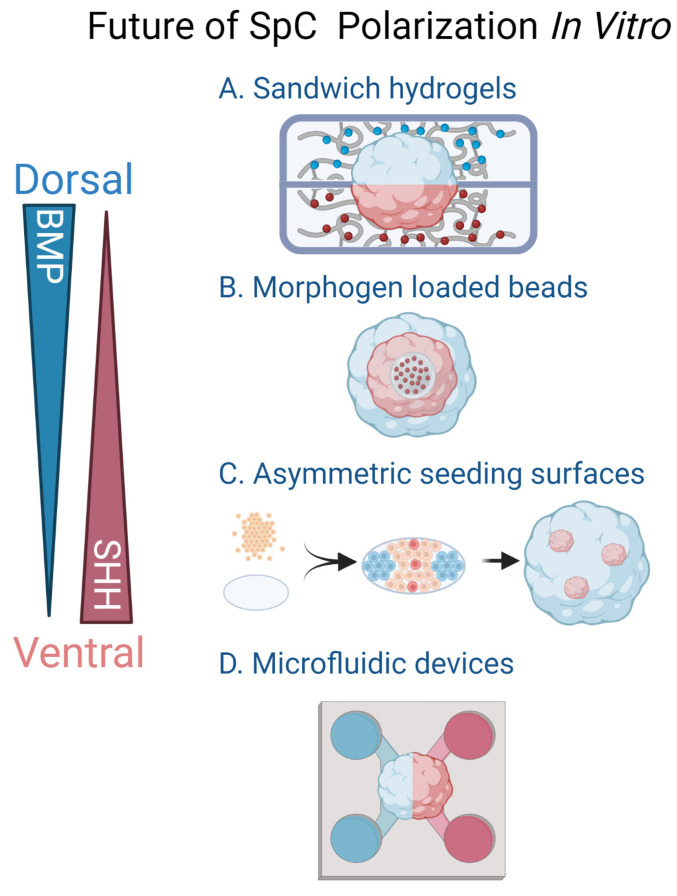
New technological approaches to induce SpC polarization in vitro. (**A**) Customized “sandwich” hydrogels loaded with BMP and SHH in two separate layers, the organoid is embedded in between both. (**B**) Organoid grown around beads loaded with SHH. (**C**) An asymmetrically patterned surface for seeding cells that induces self-organization into dorsal and ventral signaling hubs of the neural tube. (**D**) A microfluidic device to generate BMP and SHH gradients to dorsalize and ventralize the SCO. Created with BioRender.com (accessed on 2 May 2023).

An interesting approach to polarize SCOs has been reported by culturing hiPSCs in 2D in a geometrical confinement, analogous to symmetry breaking during development [223]. This method was based on previous studies demonstrating the potential of micropatterned cells to form regionalized signaling centers, resembling the embryonic organization hubs [224,225,226]. However, the SCOs shown in the study contained a prominent dorsal cell cluster expressing BMP while the ventral cluster expressed relatively low levels of SHH [223], suggesting that further optimization is required to generate both organizing cell clusters equally. Sandwich-like advanced hydrogel structures [227] or beads loaded with morphogens inserted into ECMs of choice [228] are alternative polarizing techniques that rely on the passive diffusion of molecules from the gel into the organoid and can be utilized to provide different morphogens from opposing sides. In a recent study, SAG was loaded into a porous microsphere used to culture hiPSCs in ultra-low attachment plates that formed organoids with DV polarity [229]. Although promising, further research is needed to monitor and manipulate the stability and gradience emanating from the loaded beads (Figure 4).

Microfluidic devices are a more reliable alternative to generate multiple morphogen gradients simultaneously in an automized and highly controlled manner. They have the capacity to manage the abundance of proteins and small molecules released into the culture media in picoliter to milliliter volumes [230], demonstrating their superiority compared to the passive diffusion characteristic of artificial matrices. Indeed, Demers and colleagues were able to form a neural tube slice by using a microfluidic device creating gradients of RA, SHH and BMP [121]. Using a hexagonal microfluidic system to deliver RA and GDF11, Lim and colleagues produced branchial, thoracic and lumbar spMNs from a monolayer of hiPSCs [231]. This study proved the applicability of microfluidic devices for increasing anterior–posterior heterogeneity of spMNs, albeit in a 2D culture. Microfluidic devices are also starting to be applied in MND research using 3D cultures as the main model system for drug screening purposes. Recent studies used these devices to generate gradients of drugs, such as Riluzole [232] or Rapamycin [122], and demonstrated their suitability for optimizing drug concentration and efficacy. Organoids cultured in combination with microfluidic devices are often termed organ-on-a-chip, and they have been recently been applied in the MND field to generate structures where MN somas and muscle fibers are contained in separate compartments and connected by motor axons confined into nerve bundles [118,120,233]. This procedure enabled the reliable measurement of muscle contractions that could be easily manipulated by chemical or genetic approaches, and created advanced platforms to test drugs with potential impact on axon fascicles and NMJ functionality.

As described above, the maturation and connectivity of neural circuits are key aspects of brain organoid technology that are still intensively researched to improve their resemblance of an in vivo scenario. The combination of organ-on-a-chip systems compatible with multielectrode recording are starting to be used to measure advances at this front, as well as to model electrophysiological aspects of NDs and test drug candidates. An example of the applicability of this technology can be found in a recent study where dorsal SCOs generated by following traditional small-molecule-based patterning, were transferred to a multiple electrode array chip to investigate nociceptive circuitry, and an increased electrophysiological activity was detected upon treatment with pain evoking reagents [234]. Applying organ-on-a-chip technology to the initial patterning stages of SCO formation should enable us to control the small molecule composition of the media, to create morphogen gradients in microscales and thus to polarize the SCOs in AP and DV axes while reducing manual handling that can introduce variability. Likely, if combined with a scaffolding gel that provides an ECM that nurtures neural development, such systems can yield improved SpC models that can be exploited to deepen our knowledge of development and disease.

## 8. Concluding Remarks

**Figure 5:**
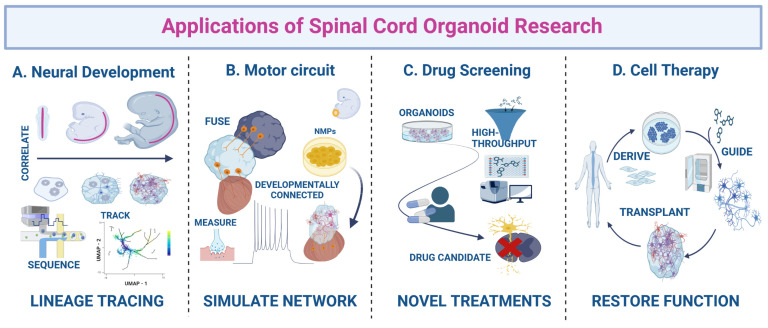
Overview of applications for spinal cord organoid (SCO) modeling. (**A**) Studying human spinal cord development by collecting SCOs at different time points allows us to perform longitudinal studies (i.e., scRNAseq analysis, lineage tracing) that can be benchmarked against in vivo counterparts. (**B**) Assembling multiple organoids or spheroids, fusing them into one coherent structure, allows researchers to study corticomotor circuits. Alternatively, hiPSCs can be induced into neuromesodermal progenitors (NMPs), bipotent cells that can give rise to both neuroectoderm and the cognate skeletal muscle. (**C**) High-throughput small-molecule screens on SCOs to identify new potential therapeutic targets. (**D**) SCOs or their derivatives have the potential to become sources for cell transplantation, aiming to ameliorate neuronal loss or restore and regenerate function in neuromuscular diseases. Created with BioRender.com (accessed on 17 May 2023).

Despite decades of research and thousands of studies, the scarcity of efficacious treatments for MNDs is obvious. This is in part due to our incomplete understanding of the molecular and cellular basis of these human diseases or the development of the human neuromuscular system. While human in vitro 2D cultures have been highly revealing to identify cell-intrinsic pathogenic factors and their response to external modulation, the numerous non-cell autonomous contributors to MND pathology are not well recapitulated in those simplified systems. More complex and controllable 3D models overcome that important limitation and enable exploring the crosstalk between the affected neurons with their muscle targets, the immunomodulatory role of glial cells or the nurturing and tissue homeostasis support provided by the vascular system. While cortical organoids have been most evolved and used to study human cortical development, in recent years, numerous SpC-like tissue models have emerged, advancing our understanding on how the SpC develops and how the neuromuscular system functions and deteriorates in pathological situations. These models include gastruloids to study the earliest phases of neural tube formation, trunk-like structures to study neural tube elongation and somitogenesis and more mature SCOs, in some cases including the muscular and cortical components of the corticomotor system, to study MNDs. Most likely, only after fully understanding the embryonic development of the human SpC, we could then explain how diseases surface. The continuous refinement of these 3D in vitro models, and the advantages they introduce to study human-specific processes in an experimentally tractable manner, hold great promise to elucidate the spatiotemporal mechanisms of SpC morphogenesis and the molecular and cellular contributors to MNDs. Constant conceptual and technological progress (i.e., adding functional vascular networks and immune cells, generating morphogen gradients, including physiologically relevant ECMs) make SCOs a powerful system to fill those knowledge gaps (Figure 5). These models provide a platform for predicting the appearance of pathological phenotypes and their response to manipulation, discovering new therapeutic candidates and testing their efficacy, potentially in a patient-specific manner. Additionally, although still far from a potential clinical application, transplantation research in murine models is evincing that genome-engineered SCOs, or their derivatives, could be a valuable source for stem cell therapy for MNDs. Overcoming the major obstacles outlined here through the application of the quickly evolving discoveries and approaches mentioned should spur the progress toward finding those highly needed therapies.

## Figures and Tables

**Table 1:** Overview of in vitro protocols for the generation of 3D spinal cord models.

Reference	Main Finding	Duration (Days)	Characterization	Cell Markers	% of spMNs	Axial Identity of Progenitors or Neurons	ECM
Meinhardt et al., 2014 [87]	First protocol showing DV-like stratification	7	qRT-PCR, IF	Floor plate cells (SHH), roof plate cells (MSX1), dorsal progenitors (PAX3, PAX7), spMNs (ISL1), pMNs (OLIG2)	Unspecified	Cervical (HOXB4/HOXC4, HOXC6)	—
Ogura et al., 2018 [88]	Titration of ventralization efficency by SAG and dorsalization by BMP4 in 3D; contains a SHH + FP-like organizer	24	qRT-PCR, IF	dI1-6 dorsal interneurons (BRN3A, LHX1, LHX9), RP cell (LMX1A), V2 interneurons (CHX10, GATA3), spMNs (ChAT + HB9 + ISL1), glia (GFAP), FP (SHH, FOXA2), dorsal progenitor cells (OLIG3, PAX7)	Unspecified	Cervical (HOXC5/HOXC6), thoracic (HOXC9)	—
Duval et al., 2019 [89]	Fine-tuning of dorsalization	14	qRT-PCR, IF	NCCs (TFAP2a), roof plate cells (LMX1B), dl1-3 dorsal interneurons (LHX1, FOXD3), dorsal progenitors (PAX7, OLIG3)	Unspecified	Hindbrain (HOXB1/HOXB4), cervical (HOXA5/HOXC6)	—
Hor et al., 2018 [90]/Hor and Ng 2020 [104]	First SCO protocol to embedded into ECM (Matrigel)	>42	qRT-PCR, IF	spMN (ISL1, ChAT, SMI32), astrocytes (S100ß), V1 inhibitory interneurons, V2a interneurons (CHX10)	55%	Cervical (HOXB4), thoracic (HOXC8)	Matrigel
Andersen et al., 2020 [79]	First SpC-spheroid protocol to form assembloid; titration Matrix for increased spMN yield (FGF2-SAG-RA)	>43	scRNAseq, qRT-PCR, IF, rabies-∆G tracing, optogenetics, Ephys	spMNs (ISL1, HB9), pMNs (OLIG2, NKX6.1), V2 interneurons (CHX10, GATA3), astrocytes (S100ß)	~45%	Cervical (HOXA2, HOXC5, HOXC6), thoracic (HOXC9)	—
Faustino-Martins et al., 2020 [100]	First SCO protocol to use NMPs; integrated skeletal muscle	>50 to 150	scRNAseq, electron microscopy, qRT-PCR, IF, microelectrode-array	spMNs (SMI32, ChAT), glia (GFAP), Schwann cells (S100β), V2a interneurons (CHX10), satellite cells (PAX7), Skeletal Muscle (TITIN, MYOD1, DESMIN), pMNs (OLIG2)	~6%	Thoracic (HOXC9), lumbar (HOXC10)	—
Mouilleau et al., 2021 [49]	SpC-spheroids pushed toward CE-THO-LU axial identity in controlled way, manipulate HOX clock	14	bulkRNAseq, qRT-PCR, IF	spMNs (ISL1, HB9)	40–80%	Cervical (HOXC6), thoracic (HOXC8, HOXC9, HOXD9), lumbar (HOXC10)	—
Pereira et al., 2021 [105]	High diversity of cell types; includes isogenic ALS hiPSC lines	>9 to 90	scRNAseq, qRT-PCR, IF, Ephys, Electronmicroscopy	NCCs (FOXD3, SOX9, SOX10, SNAI2), NSCs (SOX2), neural progenitors (OLIG3), spMNs (HB9, ChAT), microglia (IBA1, TMEM119, CX3CR1), endothelial cells (CDH5), skeletal muscle (MYF5, MYOG)	25–70%	Unspecified	Matrigel
Libby et al., 2021 [102]	Elongating gastruloids from NMPs	12	scRNAseq, bulkRNAseq, IF, whole-mount light-sheet imaging, in situ hybridization	Axial progenitors (TBXT, SOX2), neurons (ßIII-tubulin)	Unspecified	Hindbrain (HOXB1), cervical (HOXC6) and thoracic (HOXA9, HOXB9, HOXC9)	Matrigel in medium
Chooi et al., 2023 [106]	First SCO protocol to achieve high amount of ISL1 + spMNs by using alternative ECM (alginate)	>30–120	qRT-PCR, IF, Ephys, Electronmicroscopy	spMNs (ISL1, ISL2), astrocytes (GFAP), NSC (SOX2), pMNs (OLIG2), forebrain and midbrain neurons (FOXA2, FOXG1 and OTX2)	40–70%	Cervical (HOXA4, HOXB4), thoracic (HOXB8, HOXB9, HOXC8, HOXC9)	Alginate
Lee et al., 2022 [107]	First SCO protocol showing neural tube such as elongating structures, SCOs used in a high-throughput drug screen	15–140	qRT-PCR, scRNAseq, 3D imaging, IF, Ephys, electron microscopy, fuse with chick notochord, co-culture with skeletal muscle	V2a interneurons (VSX2, LHX3), spMNs (ChAT), spMNs (ISL1), V0 interneurons (EVX1), V1 interneurons (FOXP1), pMNs (NKX6.1), astrocytes (S100ß, GFAP), oligodendrocyte (MBP), dorsal progenitor cells (PAX3, PAX7, DBX1)	Unspecified	Cervical (HOXB4, HOXB7, others), thoracic (HOXC8)	Matrigel
Xu et al., 2023 [99]	High percentage of spMNs from pre-patterned caudal progenitors	28	qRT-PCR, IF, Ephys, bulkRNAseq	spMNs (HB9, ISL1), pMNs (OLIG2)	70–90%	Cervical (HOXB4), thoracic (HOXC9), lumbar (HOXD11)	—
Whye et al., 2023 [98]	Refining differentiation of spMNs from NMPs	>21	qRT-PCR, IF	spMNs (ISL1+)	Unspecified	Thoracic (HOXA9), lumbar (HOXA10, HOXA11, HOXC10, HOXD10, HOXD12)	Geltrex
Grass et al., 2023 [108]	SMA isogenic context and longitudinal study	38	qRT-PCR, IF, Live-Imaging	NSCs (SOX2, NESTIN), spMNs (HB9, ISL1, CHAT), pMNs (NKX6.1), neurons (MAP2, SMI32)	30–60%	Unspecified	Matrigel

ALS, Amyotrophic lateral sclerosis; ECM, extracellular matrix; CE, cervical; Ephys, electrophysiology; DV, dorsoventral; IF, immunofluorescence staining; FP, floor plate; HOX, homeobox; LU, lumbar; NCC, neural crest cells; NMP, neuromesodermal progenitor; NSCs, neural stem cells; pMNs, motor neuron progenitor cell; qRT-PCR, quantitative real-time polymerase chain reaction; SAG, smoothened agonist; SMA, spinal muscular atrophy; SCO, spinal cord organoid; SHH, Sonic hedgehog; SpC, spinal cord; spMNs, spinal motor neurons; THO, thoracic.

**Table 2:** 3D models to study spinal cord (SpC) development and neuromuscular diseases.

Reference	Type of Organoid	Main Findings/Achievements	Application for Modeling Disease and Development
Faustino-Martins et al., 2020 [100]	Neuromuscular organoids	NMP differentiation yields both components of neuromuscular system (spMNs and skeletal muscle)	MG, neuromuscular diseases, NMJ formation
Andersen et al., 2020 [79]	Cortico-motor assembloids	Cortical, SpC and muscle spheroids joined together to form functional cortico-motor circuits	Circuit development and degeneration in MNDs
Veenvliet et al., 2020 [92]	Gastruloid	mESCs-derived gastruloids undergo extensive morphogenesis to form neural tube and somites	Organogenesis of SpC until mid-gestation
Moris et al., 2020 [101]	Gastruloid	hESC-derived gastruloids elongate, resembling human CS9 embryos	Human-specific regulatory features of axial elongation
Pereira et al., 2021 [105]	Sensorimotor organoids	Physiologically functional NMJs are impaired in ALS	Autonomous and non-cell autonomous contributors to motor and sensory diseases
Hor et al., 2018 [90]	Ventral SCOs	Aberrant cell cycle activity in SMA spMNs	Exploring the basis of spMN degeneration in SMA
Grass et al., 2023 [108]	Ventral SCOs	First isogenic SMA hiPSC model that unravels neurodevelopmental abnormalities in SMA	Uncovering early developmental alterations in SMA
Kawada et al., 2019 [118]	Nerve organoid	Axon fascicle formation in microchannels	Study of axons grown in bundles for understanding axonal defects
Uzel et al., 2016 [119]	NMJ-on-chip	Optically excitable spMNs innervate muscles in a compartmentalized microfluidic device	Impacts of hyper/hypoactivity of spMNs on NMJ functionality
Osaki et al., 2018 [120]	NMJ-on-chip	Reduced contraction and death of muscle fibers innervated by optogenetic ALS spMNs	Investigating ALS NMJ pathogenesis and therapeutic candidates
Demers et al., 2016 [121]	NT-on-chip	A versatile microfluidic platform forming a neural tube-like sphere	Morphogen gradients to generate functional AP and DV neural tube axis
Chennampally et al., 2021 [122]	SCO-on-chip	Microfluidic device generates drug gradients to rescue ALS spMN degeneration	Small molecule gradient screens for drug concentration optimization

ALS: Amyotrophic lateral sclerosis, AP: anterior-posterior, CS9: Carnegie stage 9, DV: dorsoventral, hESC: human embryonic stem cells, mESC: mouse embryonic stem cells, MG: myasthenia gravis, MND: motor neuron disease, NMJ: neuromuscular junctions, NMP: neuromesodermal progenitor, NT-on-chip: neural tube on chip, SCO: spinal cord organoid, SMA: spinal muscular atrophy, spMN: spinal cord motor neuron.

## Data Availability

No new data were created or analyzed in this study. Data sharing is not applicable to this article.
